# Forkhead box protein 1 transcriptionally activates sestrin1 to alleviate oxidized low-density lipoprotein-induced inflammation and lipid accumulation in macrophages

**DOI:** 10.1080/21655979.2021.2000228

**Published:** 2022-01-19

**Authors:** Feng Gao, Yongcheng Zhao, Bin Zhang, Chunwei Xiao, Zhanfa Sun, Yuan Gao, Xueyong Dou

**Affiliations:** Department of Cardiovascular Surgery, Xuzhou Cancer Hospital, Xuzhou, People’s Republic of China

**Keywords:** Atherosclerosis, inflammation, lipid accumulation, FOXP1, SESN1

## Abstract

Transcription factor forkhead box protein 1 (FOXP1) has been shown cardiovascular protection. We aimed to analyze the role of FOXP1 in oxidized low-density lipoprotein (ox-LDL)-induced macrophages and its possible regulatory effect on sestrin1 (SESN1) expression. After stimulation with ox-LDL, FOXP1 expression in RAW264.7 cells was evaluated with RT-qPCR and Western blotting. Then, FOXP1 was overexpressed, followed by detection of inflammatory mediator levels using ELISA kits and RT-qPCR. Lipid accumulation was detected with oil red O staining. Additionally, the JASPAR database was used to predict the potential genes that could be transcriptionally regulated by FOXP1. ChIP and luciferase reporter assays were used to verify this combination. To further clarify the regulatory effects of FOXP1 on SESN1 in damage of macrophages triggered by ox-LDL, SESN1 was silenced to determine the inflammation and lipid accumulation under the condition of FOXP1 overexpression. Results indicated that ox-LDL stimulation led to a significant decrease in FOXP1 expression. FOXP1 overexpression notably reduced the levels of tumor necrosis factor (TNF)-α, interleukin (IL)-1β and IL-6, accompanied by a decreased in phosphorylated NF-κB p65 expression. Besides, FOXP1-upregulation inhibited lipid accumulation and reduced CD36 expression level in RAW264.7 cells upon ox-LDL stimulation. Moreover, results of ChIP and luciferase reporter assays suggested that FOXP1 could transcriptionally regulate SESN1 expression. Further experiments supported that SESN1 silencing restored the inhibitory effects of FOXP1 overexpression on the inflammation and lipid accumulation in RAW264.7 cells exposed to ox-LDL. Collectively, FOXP1 transcriptionally activates SESN1 for the alleviation of ox-LDL-induced inflammation and lipid accumulation in macrophages.

## Introduction

Atherosclerosis, the most common and serious arterial inflammatory disease that ranks the second leading cause of death with high morbidity, constitutes the pathophysiological basis of cardiovascular diseases [1–3]. Although its pathogenesis has not been fully elucidated, a large number of reports have demonstrated that inflammation and lipid accumulation mediated by macrophage activation exert crucial effects on the occurrence and progression of atherosclerotic plaques [4,5]. In recent years, despite that a lot of research on atherosclerosis has been carried out, the current treatment efficacy has not been greatly improved [6,7]. Therefore, developing effective strategies for the prevention of atherosclerosis is a top priority.

Forkhead box protein 1 (FOXP1), one of FOXP subfamily of the fox-transcription factors containing a C-terminal forkhead domain together with N-terminal zinc finger and leucine zipper domains, is first identified by Shu et al in 2001 [8]. Accumulating evidence has demonstrated the cardiovascular protection of FOXP1. For instance, FOXP1 overexpression can attenuate miR-206-induced cardiac hypertrophy [9]. It is worthy of note that FOXP1 expression is remarkably downregulated in atherosclerosis and atherosclerosis-susceptible endothelial human coronary arteries and mouse arteries [10]. Zhang et al also revealed that FOXP1 loss-of-function accelerates the development of atherosclerosis via NOD-like receptor family pyrin domain containing 3 (NLRP3) inflammasome signaling in Apoe^KO^ hyperlipidemic mouse model [10]. As an important transcription factor, the role of FOXP1 in inflammation and lipid accumulation of macrophages upon ox-LDL stimulation and the downstream genes transcriptionally regulated by FOXP1 remains unknown. By JASPAR database, we found that FOXP1 can bind to the promotor of sestrin1 (SESN1), a member of sestrin protein family. SESN1 has been demonstrated to be an atherosclerosis-related marker, and SESN1 can alleviate the release of inflammatory factors in macrophages of an atherosclerosis animal model [11].

In this study, ox-LDL-induced murine macrophage cell line RAW264.7 was used to explore the role of FOXP1 in inflammation and lipid accumulation during atherosclerosis progression. The subsequently experiments focused on whether FOXP1 can transcriptionally activate SESN1 to protect macrophages against ox-LDL-triggered damage of RAW264.7 cells. Our findings might provide novel insight into understanding the pathogenesis of atherosclerosis and identify promising targets for treatment of this disease.

## Materials and Methods

### Cell culture

RAW264.7 cells provided by the Cell Bank of the Chinese Academy of Sciences (Shanghai, China) were grown in Dulbecco’s modified Eagle’s medium (DMEM; Hyclone, USA) containing 10% fetal bovine serum (AlphaCell, China) in a 5% CO_2_ atmosphere at 37°C. When cell confluence was up to 80%, RAW264.7 cells were treated with various concentrations of ox-LDL (25, 50, 75 and 100 μg/ml) for 24 h [12].

## Cell transfection

Cells were seeded into a 96-well plate and cultured overnight for attachment. FOXP1 plasmid (Ov-FOXP1) and the empty vector plasmid (Ov-NC), small hairpin RNA (shRNA) targeting SESN1 (shRNA-SESN1-1 or shRNA-SESN1-2) and the scrambled negative control (shRNA-NC) were transfected into RAW264.7 cells by Lipofectamine® 3000 (Invitrogen; Thermo Fisher Scientific, Inc.). After 48 h of transfection, the RAW264.7 cells were harvested and the transfection efficiency was evaluated by Western blot analysis and reverse transcription-quantitative PCR (RT-qPCR) assay.

## Test for inflammatory factors contents

After the indicated treatment, the cell culture fluid was collected and the supernatant was obtained via centrifugation. Enzyme-linked immunosorbent assay (ELISA) sandwich method was employed to evaluate the concentrations of tumor necrosis factor (TNF)-α, interleukin (IL)-1β and IL-6 in culture medium supernatant in accordance with the manufacturer’s protocols. Above kits were provided by Shanghai Xitang Biotechnology (Shanghai, China). The optical density at 450 nm was determined with a microplate microscope.

## Oil red O staining

Cells cultured in 24-well sterile culture plates were fixed with 4% paraformaldehyde for 15 min. Then, cells were immersed with 60% isopropanol and stained with Oil red O solution for 10 min. After washing with phosphate buffer saline (PBS) solution, cells were counterstained with hematoxylin before cell drying. The positive macrophages were observed under the light microscope.

## Chromatin immunoprecipitation (ChIP) assay

RAW264.7 cells were crosslinked with 1% formaldehyde for 10 min and cell lysates in lysis buffer were sonicated to achieve chromatin fragments. ChIP assay was performed with a ChIP assay using a kit (Beyotime, Shanghai, China). FOXP1 antibody was adopted for the generation of immunoprecipitations and IgG antibody was used as the blank control group. The recuperated DNA fragments were determined by qPCR [13].

## Dual-luciferase reporter assay

Wild type (WT) or mutant (MUT) promoter site of SESN1 was inserted into the pGL3-Basic vector (Promega, Madison, WI) to produce WT-SESN1 promoter and MUT-SESN1 promoter plasmids. Subsequently, RAW264.7 cells were co-transfected with luciferase reporter plasmids, Ov-FOXP1 or Ov-NC by Lipofectamine®3000 (Invitrogen; Thermo Fisher Scientific, Inc.). The luciferase activity was detected by means of the Dual-Luciferase Assay System (Promega) at 48 h after transfection.

## RT-qPCR

A TRIzol® reagent (Invitrogen, Carlsbad, CA, USA) was employed to extract the total RNA from RAW264.7 cells. Complementary DNA (cDNA) was synthesized by a reverse transcriptase cDNA synthesis kit (PrimeScript™ RT Reagent Kit; Takara Bio, Inc.). qPCR was performed by specific primers on an ABI 7500 Real-Time PCR detection instrument (Thermo Fisher Scientific, Inc.). The fold difference in mRNA levels was determined using the comparative CT method (2− ΔΔCq) following normalization to β-actin mRNA [14].

## Western blot assay

RAW264.7 cells were harvested and lysed with RIPA lysis buffer (Beyotime, Shanghai, China). Afterward, 40 μg of protein was resolved by 10% sodium dodecyl sulfatepolyacrylamide gel electrophoresis (SDS-PAGE) gel electrophoresis, followed by transferred to polyvinylidene fluoride (PVDF) membranes (Millipore, Burlington, MA, USA). Blots were blocked with 5% skimmed milk, and then incubated with the appropriate primary antibodies. This was then followed by incubation with horseradish peroxidase (HRP)-conjugated secondary antibody. The gray value of protein bands was analyzed by Image J software and β-actin was used as the internal control.

## Statistical analysis

All data are shown as mean ± Standard Deviation. Statistical analysis was conducted on GraphPad Prism 8.0 (GraphPad Software, Inc., La Jolla, CA, USA). Statistical comparisons were conducted with one-away analysis of variance (ANOVA) followed by Turkey’s post hoc test. P value < 0.05 was taken as the threshold.

## Results

### FOXP1 is notably downregulated in ox-LDL-stimulated RAW264.7 cells

**Figure 1.**
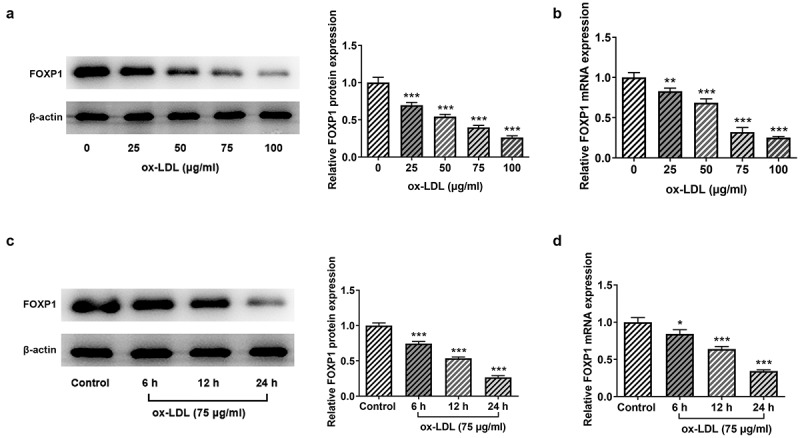
FOXP1 expression was notably decreased in ox-LDL-induced RAW264.7 cells. The (a) protein expression and (b) mRNA expression of FOXP1 in RAW264.7 cells exposed to 25, 50, 75 and 100 μg/ml ox-LDL for 24 h were analyzed with Western blotting and RT-qPCR. The (c) protein expression and (d) mRNA expression of FOXP1 in RAW264.7 cells induced with 75 μg/ml ox-LDL for 6 h, 12 h or 24 h were analyzed with Western blotting and RT-qPCR. *P < 0.05, **P < 0.01, ***P < 0.001 vs. control.

It has been reported that FOXP1 expression is remarkably downregulated in atherosclerosis and atherosclerosis-susceptible endothelial human coronary arteries and mouse arteries [10]. FOXP1 knockdown contributes to the development of atherosclerosis in a mouse model [10]. However, the expression of FOXP1 in ox-LDL-induced macrophages remains unknown. Firstly, the expression of FOXP1 after various doses of ox-LDL (25, 50, 75 and 100 μg/ml) induction was tested with Western blot analysis. As exhibited in Figure 1a-b, ox-LDL stimulation for 24 h significantly concentration-dependently downregulated FOXP1 protein and mRNA expression. Besides, 75 μg/ml ox-LDL led to remarkable decrease in FOXP1 expression in a time-dependent way at transcriptional and post-transcriptional levels (Figure 1c-d). Therefore, RAW264.7 cells stimulated with 75 μg/ml ox-LDL for 24 h were employed to perform the subsequent experiments. These data implicate an abnormal low FOXP1 expression in RAW264.7 cells after ox-LDL exposure.

### FOXP1 overexpression attenuates inflammation and lipid accumulation in RAW264.7 cells exposed to ox-LDL

**Figure 2.**
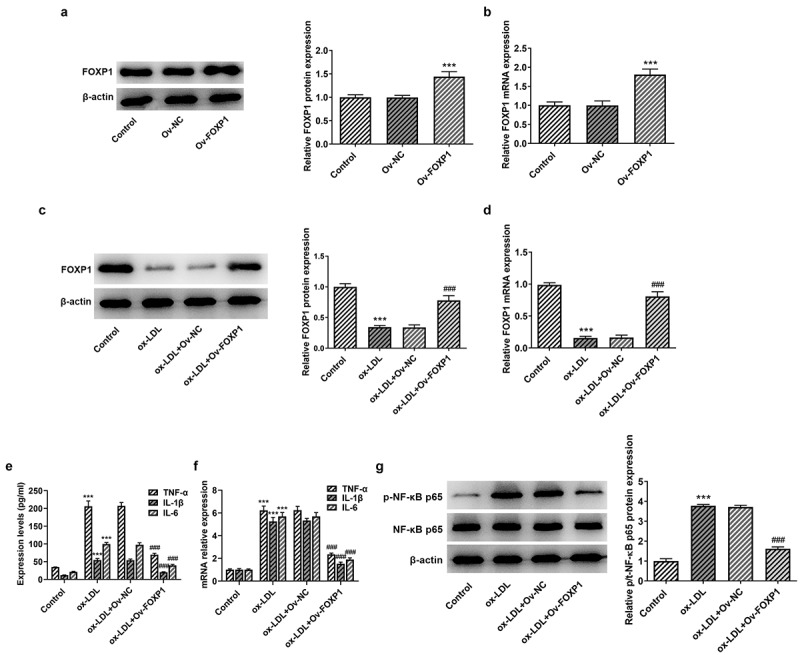
FOXP1 overexpression mitigated ox-LDL-induced inflammation in RAW264.7 cells. Measurement of FOXP1 (a) protein and (b) mRNA expression after FOXP1 overexpression in RAW264.7 cells with Western blot analysis and RT-qPCR. ***P < 0.001 vs. Ov-NC. Detection of FOXP1 (c) protein and (d) mRNA expression using Western blotting and RT-qPCR after FOXP1 overexpression in ox-LDL-stimulated RAW264.7 cells. TNF-α, IL-1β and IL-6 levels were examined by (e) ELISA kits and (f) RT-qPCR. (g) p-NF-κB p65 expression was tested with western bot analysis. ***P < 0.001 vs. control; ^###^P < 0.001 vs. ox-LDL+Ov-NC.

**Figure 3.**
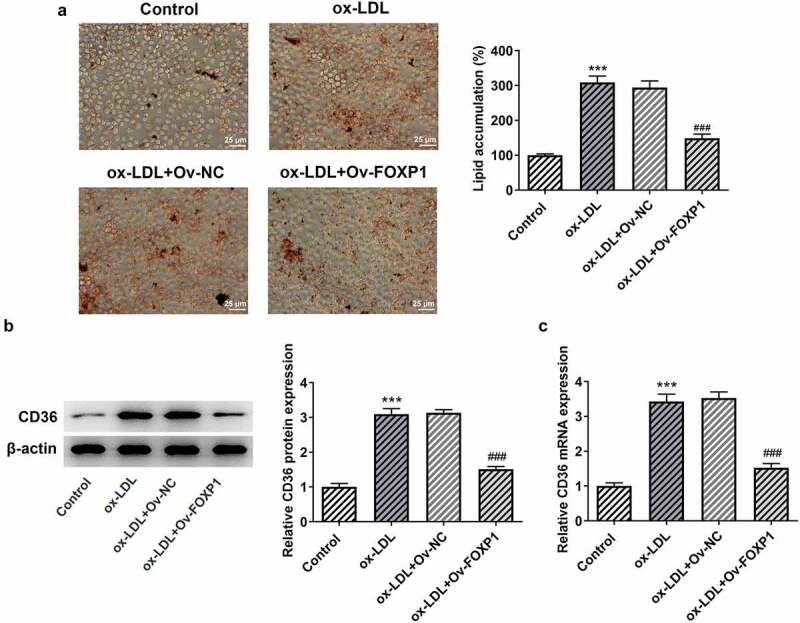
FOXP1 overexpression attenuated lipid accumulation in RAW264.7 cells challenged with ox-LDL. (a) Evaluation of lipid accumulation using Oil red O staining. CD36 (b) protein and (c) mRNA expression was measured with Western blot assay and RT-qPCR. ***P < 0.001 vs. control; ^###^P < 0.001 vs. ox-LDL+Ov-NC.

Cellular metabolism of macrophages is a manipulator of the chronic inflammation in atherosclerosis [15]. To analyze the effects of FOXP1 on the inflammation of ox-LDL-induced RAW264.7 cells, FOXP1 plasmid was transfected into cells. Significant elevated FOXP1 protein and mRNA expression was found in the Ov-FOXP1 group relative to the empty group (Figure 2a-b). Moreover, as displayed in Figure 2c-D, ox-LDL resulted in downregulation in FOXP1 protein and mRNA expression, which was dramatically restored after FOXP1 overexpression compared with the ox-LDL+Ov-NC group (Figure 2c-d). Then, results of ELISA and RT-qPCR revealed that TNF-α, IL-1β and IL-6 were conspicuously enhanced in the ox-LDL-treated group as comparison to the control group, whereas FOXP1 gain-of-function decreased the levels of these inflammatory factors (Figure 2e-f). Consistently, ox-LDL-induced upregulation of p-NF-κB p65 expression was markedly inhibited after transfection with FOXP1 overexpression plasmid as comparison to the ox-LDL+Ov-NC group (Figure 2g). Meanwhile, as what is observable from Figure 3a, the degree of lipid accumulation in RAW264.7 cells was obviously elevated compared with the control group, which was decreased by FOXP1 overexpression. The same results were found in the expression of CD36 protein and mRNA (Figure 3b-c). These results suggested that FOXP1 overexpression attenuates inflammation and lipid accumulation in ox-LDL-induced RAW264.7 cells.

### FOXP1 can bind to SESN1 promotor region and regulate SESN1 expression in ox-LDL-stimulated RAW264.7 cells

**Figure 4.**
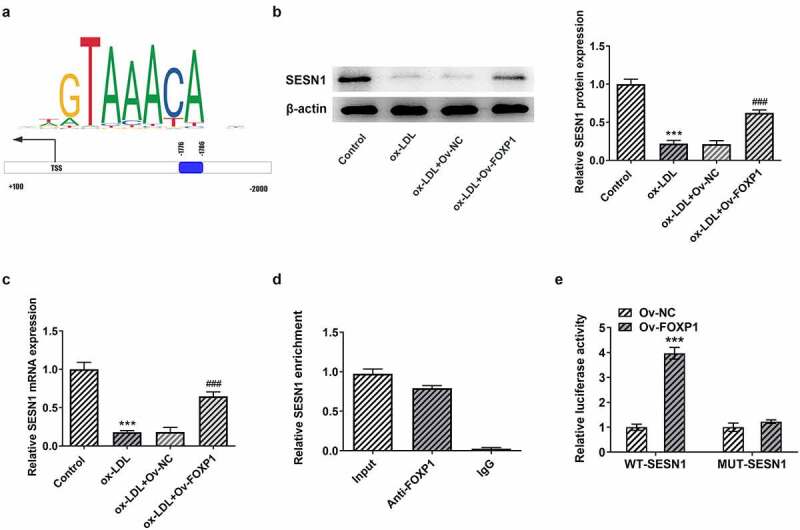
FOXP1 bound to SESN1 promotor and regulated SESN1 expression in RAW264.7 cells triggered by ox-LDL. (a) The SESN1 promotor region that FOXP1 could bind to was predicted by JASPAR database. The expression of SESN1 (b) protein and (c) mRNA after transfection with FOXP1 plasmid in ox-LDL-challenged RAW264.7 cells were tested with Western blotting and RT-qPCR. ***P < 0.001 vs. control; ^###^P < 0.001 vs. ox-LDL+Ov-NC. (d) Direct binding of FOXP1 to SESN1 promotor was verified in RAW264.7 cells by a chromosomal immunoprecipitation assay. (e) Use the dual luciferase reporter assay to check the binding of FOXP1 to the SESN1 promoter region. ***P < 0.001 vs. Ov-NC.

To explore the potential mechanism of FOXP1 in the regulation of inflammation and lipid accumulation in RAW264.7 cells under ox-LDL stimulation, JASPAR database (http://jaspar.genereg.net) was used to predict downstream genes that could be regulated by FOXP1. It has been found that FOXP1 could bind to SESN1 promotor region (−1776~-1786; Figure 4a). Then, as exhibited in Figure 4b-c, ox-LDL resulted in a significant decrease in SESN1 expression relative to the control group, whereas FOXP1 overexpression restored SESN1 expression. Further ChIP experiments displayed that compared with the IgG group, the SESN1 promoter sequence was notably enriched by immunoprecipitation with anti-FOXP1 antibody (Figure 4d). Concurrently, the luciferase activity in the FOXP1 overexpressed group was notably elevated in WT promoter site of SESN1 (Figure 4e). Together, these results provide the evidence that FOXP1 can transcriptionally activate SESN1 expression in ox-LDL-stimulated RAW264.7 cells.

### SESN1 knockdown crippled the impacts of FOXP1 upregulation on ox-LDL-triggered inflammation and lipid accumulation in RAW264.7 cells

**Figure 5.**
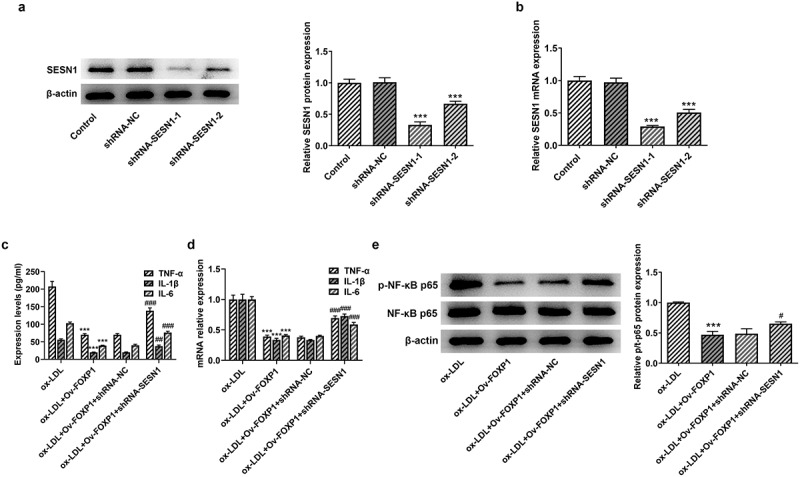
SESN1 knockdown alleviated the impacts of FOXP1 upregulation on inflammation in RAW264.7 cells induced by ox-LDL. The expression of SESN1 was assessed with (a) Western blotting and (b) RT-qPCR. ***P < 0.001 vs. shRNA-NC. Measurement of TNF-α, IL-1β and IL-6 levels using (c) ELISA and (d) RT-qPCR. (e) Detection of p-NF-κB p65 expression by Western blotting. ***P < 0.001 vs. ox-LDL; ^#^P < 0.05, ^##^P < 0.01, ^###^P < 0.001 vs. ox-LDL+Ov-FOXP1+ shRNA-NC.

**Figure 6.**
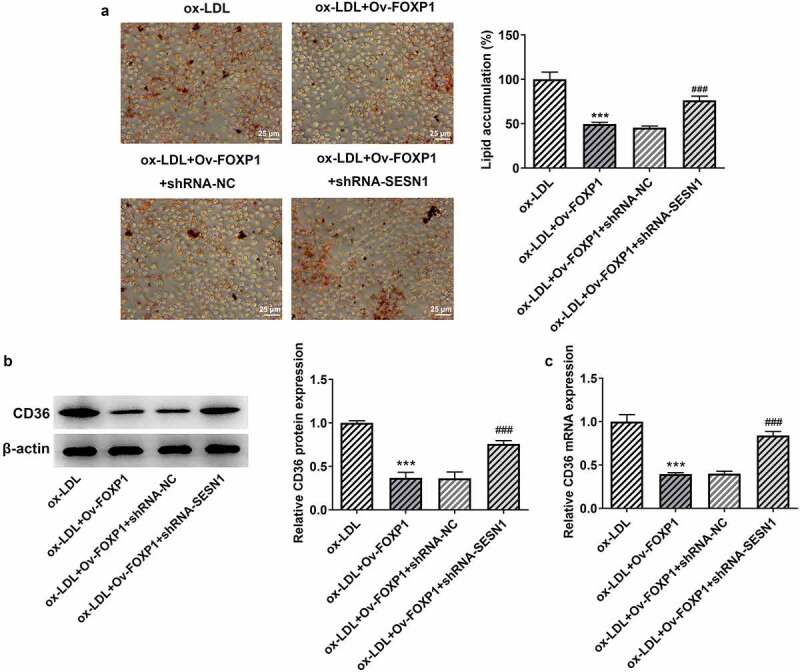
SESN1 knockdown crippled the impacts of FOXP1 overexpression on ox-LDL-triggered lipid accumulation in RAW264.7 cells. (a) Evaluation of lipid accumulation by oil red O staining. CD36 (b) protein and (c) mRNA expression was detected with Western blot assay and RT-qPCR. ***P < 0.001 vs. ox-LDL; ^###^P < 0.001 vs. ox-LDL+Ov-FOXP1+ shRNA-NC.

Afterward, whether the inhibitory effects of FOXP1 overexpression on ox-LDL-induced inflammation and lipid accumulation in RAW264.7 cells were mediated by upregulating SESN1 expression was analyzed. Firstly, SESN1 was silenced by transfection with shRNA-SESN1-1 or shRNA-SESN1-2. As displayed in Figure 5a-b, SESN1 expression was conspicuously downregulated after transfection when compared to the shRNA-NC group, and cells transfected with shRNA-SESN1-1 were used to conduct the following experiments due to lower SESN1 expression. Results of Figure 5c-d suggested significant rises in the levels of inflammatory factors TNF-α, IL-1β and IL-6 in the ox-LDL-induced RAW264.7 cells co-transfected with Ov-FOXP1 and shRNA-SESN1-1. Consistently, SESN1 silencing upregulated p-NF-κB p65 expression as comparison to the ox-LDL+Ov-FOXP1+ shRNA-NC group (Figure 5e). Additionally, the level of lipid accumulation was notably increased coupled with the upregulated CD36 expression after co-transfection with Ov-FOXP1 and shRNA-SESN1-1, relative to the ox-LDL+Ov-FOXP1+ shRNA-NC group (Figure 6a-c). Collectively, these data suggest that SESN1 knockdown reverses the impacts of FOXP1 overexpression on ox-LDL-triggered inflammation and lipid accumulation in RAW264.7 cells.

## Discussion

Macrophage-mediated inflammation and lipid accumulation are closely related to the progression of atherosclerosis, the blockade of which in macrophages may be an attractive option to alleviate this disease. We employed an ox-LDL-induced macrophage model to evaluate the roles of FOXP1 in inflammation and lipid accumulation in RAW264.7 cells. The findings in this study demonstrated that FOXP1 was notably downregulated in macrophages under ox-LDL stimulation. Further experiments in our study suggested that FOXP1 gain-of-function ameliorated ox-LDL-triggered inflammation and lipid accumulation by transcriptionally activating SESN1 expression.

FOXP1 belongs to the subfamily P of the forkhead box transcription factor family, which exerts significant effects on human normal embryonic development, cardiomyocyte development and language formation [16]. A considerable body of evidence indicates that FOXP1 has an important regulatory effect on tumor progression, such as osteosarcoma, follicular lymphoma and colorectal cancer [17,18]. In recent years, more and more scholars at home and abroad have proved the crucial role of FOXP1 in non-neoplastic diseases. For instance, FOXP1 overexpression inhibits high glucose-induced extracellular matrix accumulation and oxidative stress in mesangial cells during diabetic nephropathy [19]. Loss of FOXP1 leads to a complex cardiac phenotype characterized by defects in outflow tract septation, increased myocardial proliferation, and thinning of ventricular myocardium [20,21]. Additionally, FOXP1 is involved in the negative regulation of monocyte differentiation and macrophage function [22]. Some experts have dedicated themselves to investigating the possible effects of FOXP1 on cardiovascular diseases, and they demonstrated that FOXP1 plays crucial roles in the regulation of cardiovascular remodeling and dysfunction [21,23]. It has been also revealed that Foxp1 deletion exacerbates the formation of atherosclerotic lesion and prolongs occlusive thrombus formation [23]. Besides, downregulated FOXP1 expression has been found by Zhuang et al in atherosclerosis and atherosclerosis-susceptible endothelial human coronary arteries and mouse arteries [10]. Monocytes, which are important immune cells in atherosclerosis, gather under vascular endothelial cells and are transformed into macrophages by phagocytosis of ox-LDL [24]. Macrophages are the main source of inflammatory cytokines and the key mediator of innate immune response in all stages of atherosclerosis [25]. Macrophages exert critical effects on internalizing ox-LDL to regulate the lipid balance in the blood vessels [26]. Ox-LDL-induced inflammation and lipid accumulation in macrophages are known to be important parts of the progress of atherosclerosis [12]. As the lipids gradually accumulate in the arterial wall, macrophages absorb the excessive lipids and eventually grow into foam cells [27]. The prevention of the inflammatory response and lipid accumulation of macrophages under ox-LDL stimulation has aroused public interest in taking it as the promising therapeutic therapy for atherosclerosis. In the current study, FOXP1 expression was notably reduced in RAW264.7 cells stimulated by ox-LDL. The gain-of-function experiments suggested that FOXP1-upregulation alleviated ox-LDL-triggered inflammatory response and lipid accumulation in RAW264.7 cells.

As an important transcription factor, the downstream genes that can be regulated by FOXP1 directly were analyzed using JASPAR database, and it has been found that FOXP1 can bind to the promotor of SESN1. SESN1, a member of sestrin protein family, acts as a stress-inducible protein that modulates metabolic homeostasis upon diverse cellular stresses [28]. Yang et al have found that SESN1 plays a neuroprotective role in oxygen-glucose deprivation/reoxygenation-induced neuronal injury [29]. A significant decrease in SESN1 mRNA expression in monocytes has been revealed in serum of patients under dyslipidemic conditions [30]. In particular, SESN1 is considered as an atherosclerosis-related marker by Yang et al, and their investigation suggested that SESN1 can alleviate the release of inflammatory factors in macrophages in an atherosclerosis model [11]. In this study, SESN1 exhibited low expression in RAW264.7 cells following ox-LDL induction, which could be upregulated by FOXP1 overexpression. Results of ChIP and luciferase activity confirmed that FOXP1 could directly combine with the promotor of SESN1 to regulate SESN1 expression. The further rescue experiments indicated that SESN1 knockdown crippled the impacts of FOXP1 overexpression on ox-LDL-triggered inflammation and lipid accumulation in RAW264.7 cells, which supported the conclusion that FOXP1 transcriptionally activates SESN1 to alleviate ox-LDL-induced inflammation and lipid accumulation in macrophages.

## Conclusion

Taken together, this study describes the protective action of FOXP1 on inflammation and lipid deposition in ox-LDL-induced macrophages. Another intriguing finding in our study is that FOXP1 can transcriptionally activate SESN1 by binding to SESN1 promotor to regulate its expression, thereby relieving inflammation and lipid accumulation in macrophages under ox-LDL condition. Accordingly, FOXP1 and SESN1 might be used as promising targets for treatment of atherosclerosis.

## Data Availability

The datasets generated for this study are available on request to the corresponding author.
